# Pernicious Anæmia

**Published:** 1882-05

**Authors:** 


### PERNICIOUS ANÆMIA.

In a series of cases of pernicious anæmia Riess has found in the bone marrow an abundance of cell elements of a special character, which have rarely been met with. Besides the usual abundant colorless round cells and the nucleated red bloodcorpuscles, there were many large cellular structures containing blood-corpuscles, such as have been hitherto described only by Cohnheim, and by Gardner and Osler. They were roundish or oval cells, with a refracting clear hyaline or slightly granular stroma. Their size varied considerably; the smaller were not more than twice as large as ordinary red blood-corpuscles, while the larger were eight times as large. The colored elements which were contained in these cells also varied much in size and in number, ranging from one to twelve. When few, they resembled closely the ordinary red corpuscles, but more frequently they were smaller, darker, and more spherical, resembling the so-called microcytes. When very numerous they resembled rather fragments of red corpuscles aggregated in irregular groups. Sometimes these small elements were fused together in irregular masses. The nucleus of the containing cell was often concealed by them; and they sometimes occupied so large a part of the area of the cell that the protoplasm of the latter was reduced to a narrow circumferential zone. The number of these large cells in the bone marrow varied in different cases. Usually they were as numerous as the nucleated red blood-corpuscles, and sometimes exceeded these in number. They were found in five out of seven cases of pernicious anæmia examined. Of the two cases in which they were not found, one was not a pure case, being complicated with an affection of the kidneys, and, in both, circumstances prevented a very thorough microscopical examination. These bodies, on account of their supposed rarity, have hitherto been regarded as arising from the destruction of red blood-corpuscles. In favor of this view are the form and color of the contained corpuscles, which so often resembled fragments of corpuscles. But on this view the frequent appearance of these cells in pernicious anæma is not easy to harmonize with Neumann's theory, according to which the bone marrow in this disease is the seat of an increased formation of corpuscles. It must be assumed that there is a corresponding increase in the destruction of cells in the lymphoid medulla. In accordance with this the blood corpuscles in the marrow were found in one case to present only the aspect of microcytes, which is usually regarded as a late stage of the red corpuscles. It appears also that according to the recent observations of Grohe similar cells are to be found in the bone marrow in other diseases besides pernicious anæma.—*London Lancet.*

